# Ferroptosis and Tumor Drug Resistance: Current Status and Major Challenges

**DOI:** 10.3389/fphar.2022.879317

**Published:** 2022-05-20

**Authors:** Zhenyu Nie, Mei Chen, Yuanhui Gao, Denggao Huang, Hui Cao, Yanling Peng, Na Guo, Fei Wang, Shufang Zhang

**Affiliations:** ^1^ Central Laboratory, Affiliated Haikou Hospital of Xiangya Medical College, Central South University, Haikou, China; ^2^ Department of Urology, Hainan General Hospital, Affiliated Hainan Hospital of Hainan Medical University, Haikou, China

**Keywords:** ferroptosis, tumor drug resistance, chemotherapy, target therapy, immunotherapy

## Abstract

Ferroptosis is a novel type of regulated cell death, whose unique metabolic characteristics are commonly used to evaluate the conditions of various diseases especially in tumors. Accumulating evidence supports that ferroptosis can regulate tumor development, metastasis, and therapeutic responses. Considering to the important role of chemotherapy in tumor treatment, drug resistance has become the most serious challenge. Revealing the molecular mechanism of ferroptosis is expected to solve tumor drug resistance and find new therapies to treat cancers. In this review, we discuss the relationship between ferroptosis and tumor drug resistance, summarize the abnormal ferroptosis in tissues of different cancer types and current research progress and challenges in overcoming treatment resistance, and explore the concept of targeting ferroptosis to improve tumor treatment outcomes.

## Introduction

Malignant tumors are the second leading cause of death worldwide, following cardiovascular disease (Siegel et al., 2020). With the development of the global economy and the extension of human life expectancy, the prevention and treatment of cancer have become a global health, economic and social issue. In 2020, the number of estimated new cases of cancer and death around the world reached 1,92,92,789 and 99,58,133, respectively (Cao et al., 2021). This is equivalent to about 36.7 people being diagnosed with cancer every minute and 19 people dying from cancer. In the United States, a total of 18,06,590 new cancer cases and 6,06,520 deaths estimated to occur in 2020 (Siegel et al., 2020). The most common tumor in males is prostate cancer (21%) while that in female is breast cancer (30%). The second and third most common tumors in both sexes are lung and bronchus cancer (13% for males, 12% for females) and colon and rectum cancer (9% for males, 8% for females). Lung and bronchus cancer has the highest mortality rate in both sexes (23% for males, 22% for females) whereas colon and rectum cancer has the third highest mortality rate (9% for both); meanwhile, prostate cancer and breast cancer have the second highest mortality rate in males (10%) and females (15%), respectively (Siegel et al., 2020). China is the largest developing country. The rapid economic development has made the improvement of people’s living standards and extended the life expectancy. Cancers have become the main factor threatening the health of Chinese people. In 2020, 45,68,754 new cases of cancer were recorded in China, accounting for 48.07% of all new cases in Asia that year, and 30,02,899 deaths were noted, accounting for 51.69% in Asia (Cao et al., 2021). Drug therapy is the most important component of cancer treatment. These drugs include chemotherapeutic drugs, targeted drugs, and immune checkpoint inhibitors (ICIs). In particular, the emergence of new targeted drugs and ICIs has greatly extended the survival time of cancer patients. However, the ensuing drug resistance has become a new challenge (Balaban et al., 2004). Research on tumor drug resistance, found that many factors lead to drug resistance. Reverse or anti-drug resistance has attracted more attention in tumor research.

Ferroptosis is a kind of regulated cell death (RCD). Abnormal iron metabolism and lipid peroxidation are the two most typical characteristics in ferroptosis. Because ferroptosis is affected by the metabolism of iron ions, lipids, amino acids and other substances, a variety of ferroptosis inducers or inhibitors have been found, and the regulation of ferroptosis has been achieved *in vivo* and *in vitro*. Accumulating studies have found that the regulation of ferroptosis can affect the drug sensitivity of tumors and sensitized tumors and even reverse drug resistance. Although this discovery still requires a lot of preliminary work before clinical application, it provides new opportunities for refractory or recurring tumors. Therefore, this review summarizes the research progress of tumor drug resistance and ferroptosis to provide some ideas for either basic and clinical researchers.

## Ferroptosis

In 2003, erastin was found to induce the death of tumor cells with RAS mutations (Dolma et al., 2003). In 2008, RSL3 and RSL5 were also found to have similar efficacy to erastin, and this process can be inhibited by iron chelators or antioxidants, depending on the concentration of intracellular iron and reactive oxygen species (ROS) (Wan and Stockwell, 2008). In 2012, the term ferroptosis was to describe this cell death dependent on the accumulation of iron and lipid peroxides (Dixon et al., 2012). After nearly a decade of research, ferroptosis has been confirmed to be related to various diseases such as organ ischemia–reperfusion injury and stroke injury (Wang et al., 2019). In addition, studies have found that immunotherapy can promote ferroptosis by increasing the accumulation of lipid peroxide and iron in tumor cells, and improve the efficacy of immunotherapy (Xie et al., 2016). Ferroptosis can also affect the resistance of tumors to anti-tumor drugs, specifically through the regulation of iron and lipids.

### Mechanism of Ferroptosis

**FIGURE 1:**
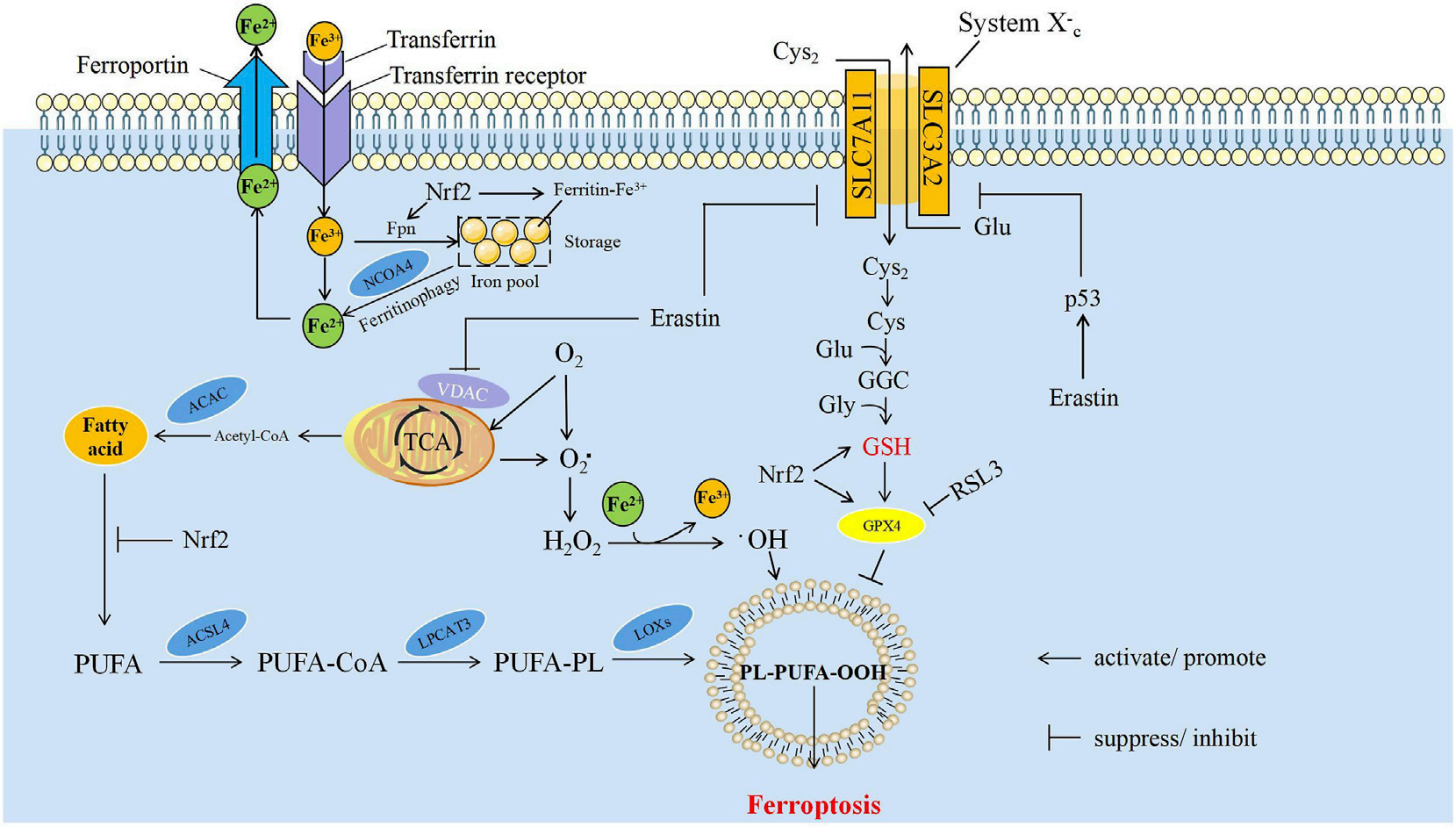
The mechanism of ferroptosis. The core mechanisms affecting ferroptosis are mainly iron metabolism, lipid peroxidation and amino acid metabolism. ACAC, acetyl CoA carboxylase; ACSL4, acyl-CoA synthetase long-chain member 4; Cys, cysteine; Cys2, cystine; Fe2+, ferrous ion; Fe3+, ferric ion; Fpn, ferroportin; Glu, glutamate; Gly, glycine; GPX4, glutathione peroxidase 4; GSH, glutathione; H2O2, hydrogen peroxide; LPCAT3, lysophosphatidylcholine acyl-transferase 3; LOXs, lipoxygenases; NCOA4, nuclear receptor coactivator 4; Nrf2, nuclear factor erythroid 2-related factor 2; O2, oxygen; O2, Oxygen free radicals, PUFA, polyunsaturated fatty acids; PUFA-CoA, polyunsaturated fatty acids-CoA; PUFA-PL, polyunsaturated fatty acids-phospholipids; PL-PUFA-OOH, phospholipids-polyunsaturated fatty acids-peroxide; SLC3A2, solute carrier family 3 member 2; SLC7A11, solute carrier family 7 member 11; TCA, tricarboxylic acid cycle.

The mechanism of ferroptosis mainly relies on intracellular dynamically balanced biochemical processes: the production and elimination of lipid peroxides. In these processes, iron and polyunsaturated fatty acids (PUFAs) are used as raw materials for lipid peroxidation to promote ferroptosis. Glutathione peroxidase 4 (GPX4) uses glutathione (GSH) as a substrate to reverse the regulation of ferroptosis. When cells cannot remove excess ROS, ferroptosis is induced. Iron, lipid, and amino acid metabolism are the most important factors in the regulation of ferroptosis (Figure 1).

#### Iron Metabolism

The participation of Fe^2+^/Fe^3+^ in the formation of ROS is one of the critical factors of ferroptosis. Iron from food is absorbed through duodenal epithelial cells. The ferric reductase in the intestinal epithelium can reduce Fe^3+^ to Fe^2+^, and then it is transported to the cells through divalent metal transporter 1 (Fleming and Bacon, 2005; Cao and Dixon, 2016). Extracellular iron can be transferred into cells by binding to transferrin and then act as a ligand for the transferrin receptor; ferroportin can transfer iron inside of cells to the outside, and then into the circulatory system (Hentze et al., 2010). Iron can be stored in ferritin which is called iron pool (IP), and it is non-toxic to the cell. Free Fe^2+^ formed labile iron pool within the cell, which is much less than the IP (Kwon et al., 2015). Free Fe^2+^ has strong reducibility and easily reacts with hydrogen peroxide (H_2_O_2_) in the cell to produce hydroxyl free radicals that can cause oxidative damage to DNA, protein, and membrane lipids; promote lipid peroxidation; and cause damage to the cell membranes, leading to cell death (Maines, 1988). The process of ferritin degradation to release Fe^2+^ is called ferritinophagy, and nuclear receptor coactivator 4 (NCOA4) acts as a linker protein to mediate this process (Mancias et al., 2014). The overexpression of NCOA4 can promote ferritinophagy, increase the concentration of free Fe^2+^ in cells, and promote ferroptosis; on the contrary, knocking down the expression of NCOA4 can inhibit the degradation of ferritin and reduce the sensitivity of cells to oxidative damage. The iron responsive element binding protein can inhibit the occurrence of ferroptosis by increasing the expression of ferritin (Dixon et al., 2012). Iron is a direct factor affecting the production of ROS. Heme oxygenase-1 catalyzes the degradation of heme and produces free Fe^2+^. The overexpression of heme oxygenase-1 can accelerate the ferroptosis induced by erastin (McKie et al., 2001; Rouault, 2005). RAS increases the intracellular iron concentration by upregulating transferrin receptor and downregulating ferritin, resulting in the occurrence of ferroptosis. RAS mutations increase the ability of cellular resistance to ferroptosis (Yang and Stockwell, 2008; Schott et al., 2015).

#### Lipid Metabolism and Lipid Peroxides

PUFAs are important components of the phospholipid bilayer in the cell membrane and maintain the fluidity. Fe^2+^ can oxidize excess PUFAs into hydroxyl free radicals through Fenton reaction, produce a large amount of lipid peroxides, and induce ferroptosis in cells (Lin et al., 2018). As a member of intracellular ROS, lipid peroxide can strongly induce ferroptosis. ROS is a class of molecules including peroxides, superoxides, singlet oxygen, and free radicals. They can cause cell death by damaging DNA, RNA and lipid molecules (Wan and Stockwell, 2015). During ferroptosis, the accumulation of lipid peroxides, especially phospholipid peroxides, is considered to be a landmark event of ferroptosis and also a prerequisite for ferroptosis (D'Herde and Krysko, 2017). Phosphatidylethanolamine (PE) is a kind of glycerophospholipid in the cell membrane. The PE content in the inner mitochondrial membrane accounts for about 40% of the total phospholipids, and it accounts for about 15%–25% in other organelle membranes (Feng and Stockwell, 2018). PE is also involved in ferroptosis induced by arachidonic acid (AA) and its derivatives (Dixon et al., 2015; Kagan et al., 2017). For example, acyl-CoA synthetase long-chain member 4 (ACSL4) can activate AA and its derivative adrenic acid into arachidene acyl-CoA and adrenal-CoA, respectively. Lysophosphatidylcholine acyl transferase 3 (LPCAT3) binds them to PE on the inner membrane, and mediates the peroxidation of membrane phospholipids through lipoxygenases (LOXs) to promote ferroptosis (Figure 1) (Sato et al., 1999; Dixon et al., 2015; Doll et al., 2016). Knockout of LOXs helps protect cells from ferroptosis induced by erastin (Yang et al., 2016).

Lipid peroxides can damage cells in many ways. First, lipid peroxides can be decomposed into ROS to amplify the lipid peroxidation process. Second, lipid peroxides can be released by changing the physical structure of the membrane, such as the thickness and degree of curvature of the membrane; perforating the membrane; releasing cytokines and disrupting the metabolism of cells. Finally, the by-products in the process of lipid peroxidation, such as malondialdehyde and 4-hydroxynonenal, can directly damage cells (Gaschler and Stockwell, 2017; van der Veen et al., 2017).

#### Amino Acid Metabolism

GSH is an important antioxidant. It can not only reduce H_2_O_2_ to H_2_O directly and maintain the balance of intracellular free radical content, but also act as a substrate of GPX4 to participate in the intracellular antioxidant system and repair cell membranes. Therefore, the activity of GSH and GPX4 is a key factor of ferroptosis (Bridges et al., 2012). Cystine/glutamate antiporter (system Xc-) is a transporter necessary for GSH synthesis. System Xc- is a heterodimer, formed by glycosylated solute carrier family 3 member 2 (SLC3A2) and non-glycosylated solute carrier family 7 member 11 (SLC7A11) linked by disulfide bonds (Gao et al., 2015). System Xc- can mediate the entry of cystine into the cell and the exchange of glutamate. Cystine can be reduced to cysteine after entering the cell, and then GSH is synthesized by the sequential activities of glutamate cysteine ligase (GCL) and glutathione synthetase (GSS) to regulate the downstream lipid peroxidation process. The imbalance of amino acid metabolism by inhibiting System Xc- can induce ferroptosis. The high concentration of extracellular glutamate can also inhibit System Xc- and induce ferroptosis. This may be one of the reasons for the cytotoxicity of glutamate in the brain (Dixon et al., 2012). Glutamine is one of the sources of glutamate. Inhibiting the decomposition of glutamine greatly improves the cell survival rate and inhibits ferroptosis in the absence of cystine (Maiorino et al., 2018). Cysteine can directly limit the biosynthesis of GSH. Therefore, cysteine can be directly transported into the cell through the alanine serine cysteine (ASC) system to inhibit ferroptosis (Doll and Conrad, 2017). Drugs, such as RSL3 (Wan and Stockwell, 2008) or hexamethylmelamine (altretamine) (Woo et al., 2015), can inhibit the expression and activity of GPX4, leading to ferroptosis (Yang et al., 2014). The content of GSH was significantly reduced in erastin-treated cells is due to the fact that erastin directly reduces uptake of cystine by inhibiting the activity of xCT (Dixon et al., 2012; Huang et al., 2018), which causes a decrease in GSH synthesis (Skouta et al., 2014; Reina and De Pinto, 2017). Therefore, the antioxidant capacity of erastin-treated cells was reduced (Reina and De Pinto, 2017).

#### Other Factors of Ferroptosis

P53 is the most widely known tumor suppressor. Under different conditions, the expression of p53 may promote, inhibit, or limit the occurrence of ferroptosis, and p53 can regulate ferroptosis from two pathways: cell-specific or environment-dependent. For example, p53 activation can inhibit the uptake of cystine by inhibiting the transcription of SLC7A11, promote the accumulation of ROS, and induce ferroptosis (Bersuker et al., 2019). The expression of the long non-coding RNA (LncRNA)-p53RRA is low in tumors. It can release p53 from the G3BP1 complex, causing the accumulation of p53 in the nucleus and ultimately inducing ferroptosis (Doll et al., 2019). It has been shown that erastin can also enhance ferroptosis by activating p53, upregulate its transcriptional product and cause an increase in ROS (Yang et al., 2014). SAT1 (spermidine/Spermine N1-acetyltransferase 1) is an important regulator in polyamine metabolism (Hu et al., 2010). In cancer cells, overexpression of SAT1 results in rapid depletion of cellular spermidine and spermine, which can inhibit cell growth and induce apoptosis (Mandal et al., 2015). It has reported that SAT1 is a transcriptional target of p53 in some cancer cell lines. Depletion of SAT1 also negatively feedback inhibits p53-induced ferroptosis in cancer cells (Ou et al., 2016). Mechanistically, SAT1 induction correlates with the expression levels of ALOX15 (arachidonate 15-lipoxygenase), and ALOX15 is a downstream effector of p53-induced SAT1 expression in ferroptosis (Ou et al., 2016). However, the deeper mechanism of p53-SAT1-ALOX15 has not yet been fully elucidated. GSL2 has been identified as a transcriptional target of p53 too, which expression is responsible for p53-mediated oxygen consumption, mitochondrial respiration, and ATP generation in cancer cells (Tarangelo et al., 2018). Since GSL2 can increase cellular antioxidant function by promoting GSH production in cancer cells, it is considered to be a negative regulator of ferroptosis (Tarangelo et al., 2018). Although p53 can induce ferroptosis through various mechanisms, surprisingly, p53 also protects cancer cells against ferroptosis through DPP4 or p21. For example, p53 can limit erastin-induced ferroptosis by blocking dipeptidyl peptidase 4 (DPP4) activity in a transcription-independent manner. Loss of p53 prevents nuclear accumulation of DPP4, thereby promoting plasma membrane-associated DPP4-dependent lipid peroxidation, ultimately leading to ferroptosis (Thomas and Thomas, 2003). The tumor suppressor p21, also known as cyclin dependent kinase inhibitor 1A (CDKN1A), is a cell cycle regulator. It has been reported that p53-mediated expression of p21 can delay the onset of ferroptosis and is associated with reduced intracellular glutathione depletion and ROS accumulation. Therefore, the p53-p21 axis may help cancer cells cope with the metabolic stress caused by cystine deprivation by delaying the onset of ferroptosis (Xie et al., 2017). In general, p53 plays an extremely important and complex role in the process of ferroptosis, and affects the occurrence and progression of ferroptosis through different signaling pathways or regulatory factors, thereby affecting tumorigenesis. A better understanding of the mechanism by which p53 controls tumor ferroptosis will help researchers discover more targets in tumor therapy.

The RAS protein plays an important role in human tumors, and its common mutation forms include KRAS, NRAS, and HRAS. Tumors with RAS mutations are sensitive to ferroptosis induced by erastin and RSL3, but the specific mechanism is unclear at present (Dolma et al., 2003; Wan and Stockwell, 2008). KRAS, which is the first reported oncogene, can regulate intracellular ROS levels through a variety of mechanisms. However, according to the erastin sensitivity analysis of 117 cancer cell lines, RAS mutations and the sensitivity of ferroptosis inducer are not significantly related compared with those in wild-type cells. This finding suggests that RAS mutations may not be sufficient as evidence of sensitivity to ferroptosis (Yang et al., 2014).

Apoptosis-inducing factor mitochondria-associated 2 (AIFM2) is also known as ferroptosis suppressor protein 1 (FSP1). It can reduce Coenzyme Q10 (CoQ10) to CoQ10H2, the latter of which acts as a radical trapping antioxidant (RTA) to quench lipid peroxyl radicals and provide protection from ferroptosis. In addition, CoQ10H2, the reduced form of CoQ10, exerts an antioxidant effect from capturing free radicals, preventing lipid peroxidation and avoiding ferroptosis (Mao et al., 2018; Shi et al., 2019).

Heat shock proteins (HSPs) are a class of highly conserved molecular chaperones that can change their expression levels in response to environmental stress, and help cells resist various types of death. Among them, HSPB1 can inhibit ferroptosis by reducing iron uptake (Sun et al., 2015). HSPA5 can bind and stabilize GPX4, avoiding the damage of lipid peroxide from ferroptosis indirectly (Yagoda et al., 2007).

Mitochondria are also involved in the occurrence of ferroptosis. CDGSH iron-sulfur domain 1 is a type of mitochondrial iron transport protein that inhibits ferroptosis by preventing the accumulation of iron in the mitochondria and the production of ROS (Yuan et al., 2016). The other is voltage-dependent anion channels (VDAC), which is a channel in the outer mitochondrial membrane that control the flow of metabolites across the membrane (Agyeman et al., 2012). Its role is to maintain mitochondrial membrane potential (ΔΨ) and to generate adenosine triphosphate (ATP). In the open state, VDAC allows the entry of the substrates of respiratory chain, adenosine diphosphate (ADP), and phosphate (Pi) into the mitochondria. Mitochondria is closed while the VDAC closed (Caterino et al., 2017). The dynamic “open-closed” state of the VDAC will have a significant impact on mitochondrial metabolism and cellular bioenergetics. Proliferating tumor cells usually have much higher levels of free tubulin (Lemasters, 2017). Low doses of tubulin can make VDAC closed and reduce ΔΨ, inhibiting the entry of respiratory substrates through VDAC into mitochondria (Maldonado et al., 2010; Maldonado, 2017). However, erastin could block the inhibitory effect of free tubulin on VDAC and promote the increase of ΔΨ and the production of mitochondrial ROS (Yagoda et al., 2007; Rostovtseva et al., 2008; Maldonado et al., 2013), and induce ferroptosis finally (Dixon et al., 2012).

The transcription factor nuclear factor erythroid 2-related factor 2 (NRF2) is an important antioxidant defense system and plays a predominantly negative role in the regulation of ferroptosis. Nrf2 acts as a transcription factor that regulates a variety of genes related to iron metabolism, lipid peroxidation and ferroptosis, such as ferritin, transferrin receptor 1 (TfR1), ferroportin (Fpn/SLC40A1), ferrochelatase (FECH), ATP-binding cassette subfamily B member 6 (ABCB6), SLC7A11, GSS, GPX4 and so on (Dodson et al., 2019). Therefore, Nrf2 can prevent ferroptosis by regulating the expression of different genes. For example, Nrf2 can reduce the unstable free iron content by upregulating the expression of ferritin (Harada et al., 2011), because both ferritin and Fpn, a key protein for iron storage, are regulated by Nrf2 (Krishnamurthy et al., 2006; Kerins and Ooi, 2018). Both ABCB6 located in the outer mitochondrial membrane and FECH in the inner membrane are positively regulated by Nrf2, which can chelate excess iron in mitochondria by promoting the synthesis of heme, reducing mitochondrial iron overload (Wu et al., 2001; Mazure, 2017). The GSH-GPX system is an important component of anti-lipid peroxidation (Yagoda et al., 2007), and Nrf2 is not only a transcription factor for GSH reductase and GPX4, but also promotes GSH biosynthesis, and prevents ferroptosis (Dodson et al., 2019). Nrf2 can also regulate the transcription of aldehyde-ketone reductase 1C1-3 and acetaldehyde dehydrogenase 3A1 (Zakharova et al., 2018), degrade the products of lipid peroxidation, and prevent the occurrence of ferroptosis (Dodson et al., 2019).

## Tumor Drug Resistance

In the past few decades, the treatment of tumors has been greatly advanced. From the earliest surgery or chemotherapy, it has gradually evolved into “polytherapy” which includes surgery, chemotherapy, immunotherapy, and molecular targeted therapy. With the proposal and wide application of polytherapy, the survival of patients has remarkably increased. Unfortunately, tumor drug resistance greatly limits the long-term survival of patients (Balaban et al., 2004).

The concept of drug resistance first came from the resistance of bacteria to antibiotics (Blakely et al., 2015; Fisher et al., 2017; Chatterjee and Bivona, 2019). In oncology, drug resistance refers to the heritable ability of cells to survive exposure to the high concentrations of a drug. “Tolerance and persistence” are concepts used to describe the sensitivity and resistance of cells to drug treatments. “Tolerance” is the ability of cells to survive ephemeral exposure to therapeutic concentrations of drugs, and “persistence” is the ability of a sub-population of cell clones to survive in a treatment (Holohan et al., 2013; Holden, 2015).

Tumor resistance to therapeutic drugs is divided into primary resistance and acquired resistance. Primary resistance refers to the phenomenon of resistance to drugs in the initial treatment, and without tumor shrinkage or remission after receiving the treatment. It is usually due to the genetic mutation or abnormal cell status in the tumor or is caused by the rapid adaptation of the cell to the drug (Konieczkowski et al., 2014; Lee et al., 2014). Compared with primary resistance, acquired resistance is more prevalent. It refers to resistance developed during treatment. At present, there are two main speculations about the mechanism of acquired resistance: pre-existing and re-evolutionary. The pre-existing hypothesis believes that there are multiple potential clonal heterogeneities in the tumor, and the rare subclones may be resistant to drugs before treatment begins. After receiving drug treatment, these resistant subclones continue to expand, eventually resulting in drug-driven tumor recurrence (Nikolaou et al., 2018; Yoda et al., 2019). The re-evolution hypothesis refers to the cell survival through epigenetic adaptation in the initial treatment, so that phenotypes such as drug resistance and cell cycle delay are developed before permanent drug resistance. This part of the cells can turn into the state of “persistence” to escape the efficacy of drugs and serve as “seeds” from which drug-resistant clones evolve (Kobayashi et al., 2005; Pao et al., 2005; Bean et al., 2007; Yun et al., 2008; Wang et al., 2015; Flinders et al., 2016; Hata et al., 2016; Li et al., 2016). It also reveals the mechanism of minimal residual disease causing tumor recurrence. The tumor microenvironment (TME) can also promote acquired resistance: the growth factors secreted by tumor cells or tumor-resident stromal cells can maintain tumor cell survival in the initial treatment (Straussman et al., 2014; Wilson et al., 2014; Obenauf et al., 2015).

### Mechanism of Tumor Drug Resistance

#### Tumor Heterogeneity

**FIGURE 2:**
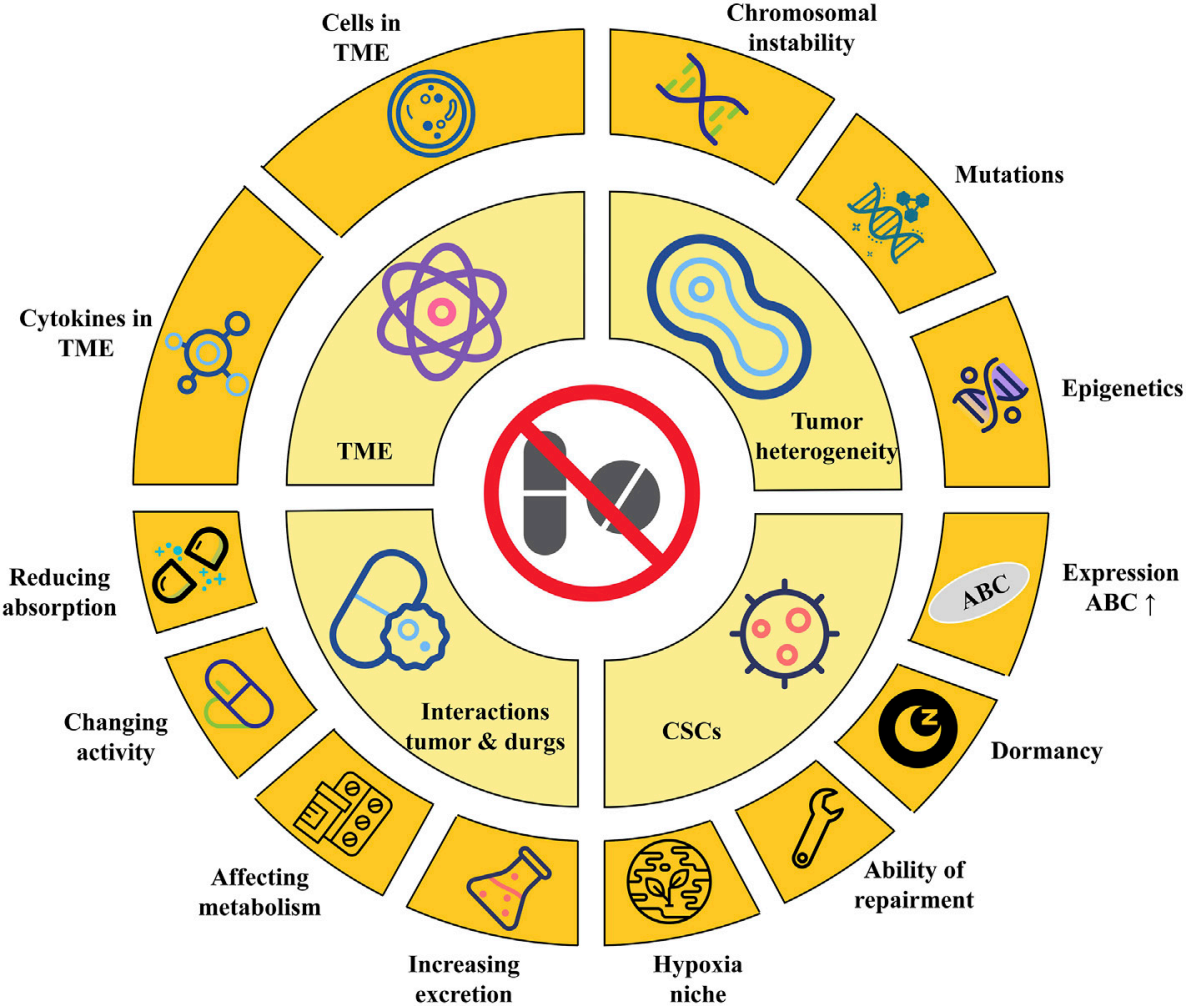
The Mechanisms of tumor drug resistance. Tumor heterogeneity is usually caused by chromosomal instability, mutations and epigenetics changes. Cancer stem cells can resist drugs by increasing the expression of ABC, dormant by arresting cell cycle, improving repair ability, and creating a hypoxic niche; The components of TME, including cells and cytokines, can cause tumor cells to resist drugs. Last but not least, tumors can resist drugs by reducing absorption, changing activity, affecting metabolism and increasing excretion. ABC, adenosine-triphosphate binding cassette; CSCs, cancer stem cells; TME, tumor micro environment.

The mechanism of tumor resistance is diverse and complex. As tumors are generally considered to be genetic diseases with genetic diversity and selective evolution, drug resistance is usually also caused by genetic changes. Hence, drug resistance, whether primary or acquired, is a definite and irreversible phenomenon (Stratton et al., 2009; Greaves and Maley, 2012; Velculescu et al., 2013). Even in the same kind of tumor, the phenotype of the tumor is different due to the different genotypes of its internal cells. This phenomenon is called tumor heterogeneity. Tumor heterogeneity is the most common factor that promotes tumor resistance (Concannon et al., 2015; Kobayashi et al., 2016; Vinogradova et al., 2016). Tumor heterogeneity also changes with the progress of tumor treatment. Studies comparing the primary tumor and metastasis found changes in heterogeneity. This phenomenon indicates that the heterogeneity of the tumor has time and spatial specificity, and it also plays a key role in tumor drug resistance (Gerlinger and Swanton, 2010). Tumor heterogeneity results from genetic or epigenetic changes in tumor cells or cells in the TME (Gorre et al., 2001; Stratton et al., 2009). Chromosomal instability, genetic missense mutations, or epigenetic changes, such as DNA methylation or histone modification, can all lead to changes in tumor heterogeneity (Figure 2) (Gorre et al., 2001; Stratton et al., 2009; Negrini et al., 2010). In chronic myeloid leukemia (CML), heterogeneity has been confirmed to cause drug resistance. BCR-ABL translocation is an established driver mutation in CML. The subclone of leukemia cells carrying imatinib-resistant mutations in the kinase domain of BCR-ABL can cause CML patients to relapse after receiving imatinib treatment (Charles, 2012; Wilting and Dannenberg, 2012). Tumor heterogeneity also promotes the resistance of lung cancer to epidermal growth factor receptor (EGFR) tyrosine kinase inhibitors (TKIs). More than 75% of non-small cell lung cancer (NSCLC) patients have multiple subclonal driver mutations, which affect the response of high-grade lung cancer to EGFR treatment (Blakely et al., 2017; Jamal-Hanjani et al., 2017). In addition, the EGFR T790M subclone can not only resist first- and second-generation EGFR inhibitors, but also promote resistance to EGFR inhibitors in NSCLC patients who are treated for the first time (Piotrowska et al., 2015).

#### Cancer Stem Cells (CSCs)

CSCs are cell subclones that have the ability to self-renew in tumor tissues and produce a series of heterogeneous tumor cells (Al-Hajj et al., 2003). CSCs are also considered to be the source of tumor heterogeneity (Bonnet and Dick, 1997). CSCs have strong abilities of proliferation and differentiation, which are similar to stem cells. In addition, CSCs have similar characteristics to side population (SP) cells. SP cells are a group of cells that deviate from the main peak in the flow cytometer scatter diagram because they are less stained by Hoechst 33,342 (Cao et al., 2011). CSCs and SP cells can excrete chemotherapeutic drugs out of cells by highly expressing adenosine triphosphate-binding cassette (ABC) G2 (Clarke et al., 2006). CSCs have stronger tumor-forming ability in *in vitro* and *in vivo* experiments; for example, about 500–1,000 CSCs cells are needed to form tumors (Jang et al., 2012; Teshima et al., 2014; Peiris-Pagès et al., 2016).

The development of drug resistance is closely related to the biological characteristics of CSCs. PAXC-002 and PAXC-003 are two colon cancer cell lines established from a primary human xenograft model. Compared with PAXC-003, PAXC-002 shows significant resistance to gemcitabine and also has strong abilities to clone and express cluster of differentiation 133 (Cioffi et al., 2015). Breast cancer MCF-7 cells pretreated with paclitaxel can obtain a stronger ability to form cell spheres, indicating that paclitaxel has a certain enrichment effect on breast CSCs (Jang et al., 2012).

The specific mechanisms of drug resistance caused by CSCs be initiated as follows (Figure 2): 1) CSCs generally highly express ABC, which can transport drugs outside the cell, reduce the intracellular drug concentration, and reduce the cytotoxic effect of the drug. Inhibiting the expression of ABC transport protein or knocking out ABC G2 gene can improve the sensitivity of CSCs to chemotherapeutics (Mare et al., 2013). 2) CSCs are mostly dormant: Given that tumor cells are usually in an active state of proliferation, irreversible damage can be inflicted on dividing cells by interfering with or inhibiting DNA or RNA synthesis, inserting alkylation, or inhibiting key enzymes required for DNA synthesis. However, most CSCs. are in the G0 phase; they are not sensitive to the efficacy of these drugs. Instead, the drug eliminates tumor cells and enriches CSCs. In pancreatic CSCs, the cyclin-dependent kinase inhibitor P21 and tumor factor P53 are highly expressed, while the expression of cyclin D1 is reduced. The cell cycle is arrested in the G0/G1 phase, and the sensitivity to chemotherapeutic agents is significantly reduced (Meng et al., 2014). In ovarian cancer, CSCs with positive acetaldehyde dehydrogenase 1 (ALDH1) expression are significantly resistant to chemotherapeutic agents. This is because ALDH can oxidize aldehydes into carboxylic acids and resist the cytotoxic effects of alkylating agents. ALDH1 can also make more cells stagnate in the G0 phase by regulating the cell cycle (Hu et al., 2012). 3) CSCs show strong repair ability in the process of coping with damage. CSCs will activate p53, ataxia-telangiectasia mutated-cell cycle checkpoint kinase 2, and other damage repair pathways, causing cell cycle arrest and providing sufficient time and opportunity for damaged cells to complete self-repair (Karran, 2001). Compared with ordinary tumor cells, CSCs highly express endonucleases, DNA polymerases, and DNA ligases related to DNA repair and have stronger enzymatic activity (Shiotani et al., 2006). 4) The special hypoxia niche in which CSCs are located helps to avoid the efficacy of drugs. Using trastuzumab to treat breast cancer can enrich breast CSCs. The interleukin-6 (IL-6) secreted by breast CSCs is about 100 times higher than that in parental cell. The inflammatory niche activated by IL-6 is the key for HER2^+^ cells to resist trastuzumab. This suggests that the cytokine of the TME also plays an important role in tumor drug resistance (Bhowmick et al., 2004).

#### Tumor Micro Environment

The TME is the environment in which tumor cells grow; it is usually composed of tumor cells, immune and inflammatory cells, tumor-associated fibroblasts, blood vessels, extracellular matrix, and various cytokines (Dobbs et al., 2016; Ireland et al., 2016). The metabolism of tumor tissue affects the changes of TME. At the same time, the TME can also reshape tumors, such as affecting tumor proliferation, invasion, metastasis, or apoptosis. Similarly, many components in the TME can also affect the sensitivity of tumor cells to drugs (Figure 2). IL-10 secreted by tumor-associated macrophages (TAMs) causes breast cancer to resist paclitaxel through the signal transducer and activator of transcription 3/B-cell lymphoma-2 pathway (Korkaya et al., 2012). In addition, TAMs can activate insulin-like growth factor (IGF) receptors on pancreatic cancer cells by secreting IGF-1 and 2, causing pancreatic cancer to resist gemcitabine (Shiao et al., 2011). The fat cells in the TME can increase the resistance of breast cancer to chemotherapy by releasing IL-6 (Yang et al., 2015). Various cytokines in the TME can activate signal pathways that maintain survival and make tumor cells resistant to chemotherapy and molecular targeted therapies. For example, in Burkitt’s lymphoma, the use of doxorubicin treatment can cause the thymus to release IL-6 and tissue inhibitor of metalloproteinase 1 (TIMP1), causing the remaining lymphoma tissue to resist the drug, which leads to the recurrence of lymphoma (Gilbert and Hemann, 2010). Hypoxia is the most common feature in the TME (Kumar and Vaidya, 2016). Hypoxia can cause cell cycle arrest in tumor cells, and can also induce an increase in P27 expression, arrest cell division in the G1/S phase, and make tumor cells resistant to chemotherapy drugs (Mazumdar et al., 2009). In cholangiocarcinoma, hypoxia can promote the expression of procollagen lysine-2-ketoglutarate 5-dioxygenase, a kind of collagen-modifying enzyme that can cause resistance to gemcitabine and promote epithelial mesenchymal transition (EMT) (Yeo et al., 2017).

#### Interactions Between Cancer and Drugs

Tumor cells can resist chemotherapy drugs by changing the activity, reducing absorption, increasing efflux, or influencing metabolism (Figure 2). For example, cytarabine needs to be phosphorylated to be converted into cytarabine triphosphate to exert anti-tumor effects. The downregulation or mutation of enzymes involved in this pathway can reduce the efficacy of cytarabine, leading to tumor cell resistance to cytarabine (Kort et al., 2015). P-protein is a multi-drug membrane transporter that can transport a variety of chemotherapeutic drugs such as doxorubicin, vincristine, or paclitaxel from the cell to the outside, thereby reducing the intracellular drug concentrations and reducing the therapeutic effect (Cros et al., 2009). Mutations in the folate carrier gene in patients with acute lymphoblastic leukemia can reduce the binding of methotrexate to transporters and cause resistance to methotrexate (De Andres and Llerena, 2016). The mechanism of chemotherapy drugs mainly depends on the metabolism of enzymes to exert anti-cancer effects, so enzymes are the most important factor in determining drug concentration. In pharmacology, drug metabolism reactions are mainly divided into phase Ⅰ and phase Ⅱ reactions. Phase Ⅰ reaction includes oxidation, reduction, and hydrolysis reactions, and phase Ⅱ reaction is a combination reaction (Guengerich, 1997). In breast cancer, increasing the activity of P450 can enhance the cell metabolism and inactivate docetaxel. Therefore, reducing the activity of cytochrome P450 can improve the therapeutic effect (Hentrich and Barta, 2016). In phase Ⅱ reaction, the drug can combine with GSH to reduce drug activity and electrophilicity and increase GSH and glutathione transferase activity, reducing anti-tumor effects of alkylating or platinum-containing chemotherapeutic agents (Liu et al., 2006).

The treatment of tumors has evolved from chemotherapy or radiotherapy to polytherapy including surgery, chemotherapy, radiotherapy, targeted therapy, immunotherapy, and other methods. Therefore, the mechanisms of tumor drug resistance are constantly being developed and improved. For example, immunotherapy is a novel remedy of treating tumors. ICI therapy uses the abnormal characteristics of tumors to find suitable targets and accurately destroy tumor cells. Drugs such as programmed cell death protein-1 (PD-1) monoclonal antibody and cytotoxic T lymphocyte-associated antigen-4 monoclonal antibody have been developed (Amaral et al., 2017). However, with the widespread application of immunotherapy, the number of patients with acquired resistance to ICIs has gradually increased. This phenomenon is related to the loss of function of T cells, the inability to form new antigens, genetic and epigenetic changes in presentation and processing, changes in the pathways of cell signaling that disrupt the action of cytotoxic T cells, and the formation of tumor immune escape cell lines (Gajewski et al., 2013; Jenkins et al., 2018). Under the selection of drugs, tumors can evolve innate and adaptive abilities in order to escape the immune system, thereby resisting the therapeutic effects of ICIs (Perrier et al., 2020). The drug resistance mechanism of tumor cells is very complicated, and this concept is constantly developing. A profound understanding of the mechanism of tumor resistance can enhance the therapeutic efficacy and benefit the survival of tumor patients. In terms of pathology, oncology is developing rapidly at present, and finding more accurate drug targets has become a new direction for the treatment of tumor drug resistance. Accumulated evidence show that the regulation of ferroptosis can promote cell death or maintain cell survival. This suggests that the regulation of ferroptosis can improve tumor drug resistance, and it will bring novel development opportunities for tumor treatment.

## Crosstalk Between Ferroptosis and Tumor Drug Resistance

**FIGURE 3:**
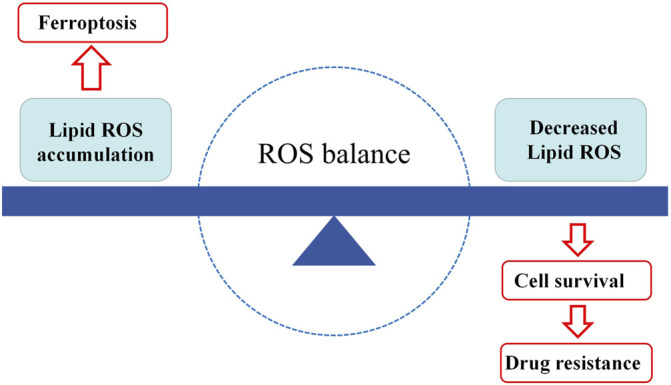
The balance of ferroptosis and drug resistance. When lipid peroxides accumulate in the cells, hence ferroptosis occurs. When lipid peroxides are decreased in the cells, the cells will have more opportunities to survive, in the result of tumor drug resistance. ROS, reactive oxygen species.

Tumor drug resistance is related to a variety of mechanisms, among which the destruction of redox homeostasis is one of the key factors leading to it. Tumor cells can enhance their tolerance to oxidative stress by inhibiting their own ROS generation, and develop acquired drug resistance (Yang et al.). Ferroptosis is closely related to the production of oxidative stress and ROS. The regulation of oxidative stress can cause changes in ferroptosis, thereby affecting the sensitivity of tumor cells to drugs. The relationship seems to be placed on two ends of a balance: on the one hand, lipid peroxidation and ROS accumulation cause ferroptosis in tumor cells, which limits tumor proliferation. On the other hand, if lipid peroxidation and ROS are reduced, tumor cells will survive and develop resistance to anti-tumor drugs. The central point is the dynamic balance between the generation and removal of ROS (Figure 3).

### Crosstalk Between Ferroptosis-Related Oxidative Stress and Tumor Drug Resistance

Increasing evidence show that abnormal iron metabolism, especially iron overload, is closely related to tumors. On the one hand, iron overload in tumor cells can catalyze the production of ROS, which can meet the needs of their proliferation to a certain extent; on the other hand, when tumor cells are exposed to drugs, they induce large amounts of ROS production, and the excessive accumulation of ROS brings great challenges for the survival of tumor cells. Once tumor cells activate a certain mechanism to change their metabolic microenvironment and inhibit the formation of ROS, drug resistance is developed (Chen et al., 2016). For example, the treatment of cervical cancer cells with deferoxamine (DFO) can enhance their sensitivity to oxaliplatin, indicating that iron overload is also a factor that regulates tumor resistance (Galadari et al., 2017). The production of ROS and ferroptosis caused by iron-triggered oxidative damage are closely related to the drug resistance of tumors (Watson, 2013). For example, oxidative stress related to ferroptosis is also an important factor in the resistance of pancreatic cancer to gemcitabine. In view of the core position of gemcitabine in the treatment of pancreatic cancer, many scholars have made efforts in this field. Nrf2 is commonly believed to play a key role in this process (Yang et al., 2021b).

Nrf2 is an important antioxidant defense system, and it plays a negative role in the regulation of ferroptosis. In liver cancer, the p62–Keap1–Nrf2 signaling pathway can inhibit ferroptosis induced by erastin and sorafenib (Sun et al., 2016b). Similarly, in NSCLC, the activation of the Keap1–Nrf2 signaling pathway can comprehensively improve the resistance of tumor cells to radiotherapy, chemotherapy, and TKIs, and is significantly associated with a shorter overall survival in patients (Dempke and Reck, 2021). Inhibiting the Keap1–Nrf2 pathway can promote ferroptosis of head and neck tumor cells and reverse the phenomenon of cisplatin resistance (Roh et al., 2017). The resistance of head and neck tumors to GPX4 inhibitors is due to the activation of the Nrf2 signaling pathway (Shin et al., 2018). iASPP is a P53 inhibitor that suppresses the ubiquitination and degradation of Nrf2, increases its accumulation, and reduces the production of ROS. It has been shown to promote tumor proliferation and increase tumor resistance to drugs *in vitro* and *in vivo* (Chen et al., 2017). ARF is an alternative reading frame product of the CDKN2A locus, which can inhibit tumors by inhibiting the negative regulators of MDM2 and ARF-BP1 (HUWE1), E3 ubiquitin ligase, and TP53 (Sherr, 2006). In addition, ARF can activate the expression of SLC7A11 by inhibiting the transcription of Nrf2, promote ferroptosis by regulating the sensitivity of tumor cells to oxidative stress, and suppress the resistance to drugs (Ge et al., 2017). In liver cancer, the expression level of Nrf2 determines the sensitivity to ferroptosis-targeted therapy (Sun et al., 2016a). Sorafenib, which is widely used to treat liver cancer, is a strong inducer of ferroptosis. The activation of the Nrf2 pathway in hepatocellular carcinoma can up-regulate the expression of metallothinonein-1G, and promote the resistance to sorafenib by inhibiting ferroptosis (Xie et al., 2016). In fact, the overexpression of Nrf2 can produce resistance to all major classes of chemotherapeutic drugs, such as anthracyclines, mitomycin, platin, antitubulin agents, vinca alkaloids, and cyclophosphamide, and even sexual hormone drugs. This phenomenon is related to the positive regulation of human aldo-keto reductases (AKRs) by Nrf2. AKRs directly participate in drug metabolism or eliminate the cell stress caused by drugs, especially ROS and lipid peroxidation, and promote tumor resistance to these drugs (Penning et al., 2021).

### Influencing Tumor Resistance by Regulating the Sensitivity to Ferroptosis

Given that ferroptosis is closely related to a variety of biochemical processes, the sensitivity of tumor cells to ferroptosis can be regulated by intervening in these key processes. For example, erastin significantly enhances the anti-tumor activity of cytarabine and doxorubicin in acute myeloid leukemia (AML) (Conrad et al., 2016). The drug resistance of AML cells can be reversed by inducing ferroptosis (Lu et al., 2017). Kidney cancer, B-cell lymphoma and triple-negative breast cancer are strongly dependent on the expression of GPX4 and xCT (Yu et al., 2015). The tumor suppressor effect of P53 is related to the inhibition of xCT on the cell membrane and induction of ferroptosis in tumors. According to the classic tumor suppressor effect of P53, ferroptosis may play a key role in the occurrence of tumors (Drayton et al., 2014).

Many ncRNAs are also involved in the regulation of ferroptosis. For example, the aforementioned lncRNA P53RRA participates in ferroptosis by regulating the expression of P53, mediating tumor resistance to drugs (Doll et al., 2019). MiR-27a reverses the resistance of bladder cancer cells to cisplatin through targeted regulation of SLC7A11 (Jiang et al., 2015). In oral squamous cell carcinoma, miR-375 can inhibit SLC7A11 transcription and regulate tumor resistance (Wu et al., 2017).

### Ferroptosis and Tumor Resistance to Cisplatin

After treatment of a variety of tumor cells with cisplatin, apoptosis and ferroptosis could occur at the same time. The currently known mechanisms of drug resistance can all affect cisplatin-induced apoptosis, but no effect has been found on cisplatin-induced ferroptosis (Guo et al., 2018). Hence, ferroptosis is different from apoptosis, which provides a new idea for solving the problem of cisplatin resistance. After pretreatment of cisplatin-resistant ovarian cancer cells with erastin, the sensitivity to cisplatin is increased, and it shows a good synergistic effect between erastin and cisplatin (Sato et al., 2018; Cheng et al., 2021). In cisplatin-resistant gastric cancer, the high expression of ATF3 can cooperate with erastin to inhibit the Keap1–Nrf2–xCT pathway, promote the ferroptosis of cancer cells, and reverse cisplatin resistance (Fu et al., 2021). However, cisplatin is not always sensitive to ferroptosis inducers in cancer cell lines. Usually, RAS mutations cause iron overload, making cells more sensitive to ferroptosis inducers (Steuer et al., 2016). About 20% of human tumors have activating mutations in RAS, and they are usually associated with drug resistance (Yang and Stockwell, 2008; Young et al., 2013). Studies have found that transient exposure of cells to low erastin concentrations can produce strong and long-lasting inhibition of xCT, leading to GSH depletion. Importantly, short-term exposure of tumor cells to erastin strongly enhances the cytotoxicity of cisplatin, thereby effectively eradicating tumor cells, which may provide guidance for cisplatin combined with erastin to treat tumors (Sato et al., 2018). Considering that cisplatin exhibits good anti-tumor efficacy in a variety of tumors, the therapeutic method of cisplatin combined with induction of ferroptosis has great potential. Moreover, it can be used to develop new ways in reversing drug resistance and expanding the application of classic therapy.

### Ferroptosis and Resistance to Targeted and Immunotherapy

The prevalence and effectiveness of molecular targeted therapy have been widely recognized. Evidence shows that ferroptosis can prevent acquired resistance to multiple targeted therapies, such as: lapatinib, erlotinib, trametinib, dabrafenib, and vemurafenib (Hangauer et al., 2017; Viswanathan et al., 2017; Tsoi et al., 2018). These targeted drug-resistant tumor cell lines express EMT markers and become more sensitive to ferroptosis (Hangauer et al., 2017; Viswanathan et al., 2017; Tsoi et al., 2018). EMT means that epithelial cells lose their characteristic polarity and adhesion ability, and acquire the ability of migration and invasion related to the mesenchymal phenotype (Yang et al., 2021a). EMT has been proven to make tumor cells resistant to drugs, which is stimulated by transcription factors, such as TWIST1 and ZEB1 (Yang et al., 2021a). For example, β-elemene can increase the sensitivity of KRAS mutant colorectal cancer cells to cetuximab by inducing ferroptosis and inhibiting EMT (Chen et al., 2020). Therefore, ferroptosis can not only regulate tumor resistance to targeted drugs, but may also be closely related to EMT.

PD-1 and PD-L1 targeted agents have been approved by the Food and Drug Administration for the treatment of a variety of tumors. Anti-PD-L1 antibody can promote lipid peroxide-dependent ferroptosis in tumor cells, and the use of ferroptosis inhibitors can inhibit the anti-tumor efficacy of anti-PD-L1 antibody. The combination of anti-PD-L1 antibody and ferroptosis inducers has been inferred to greatly inhibit tumor growth (Liu J. et al., 2018). The mechanism is related to the release of interferon-γ by cytotoxic T cells, which activate STAT1 and inhibits xCT expression, resulting in the promotion of ferroptosis (Liu J. et al., 2018). The use of ferroptosis inducers that activate STAT1 can effectively prevent tumor resistance to immunotherapy and provide a broad prospect for the clinical application of ferroptosis inducers and ICIs.

## Future Prospects

Ferroptosis responds to a complex regulatory network, including epigenetic, pre-transcriptional, post-transcriptional, and post-translational modification (Praticò and Sung, 2004). Because tumor cells are usually particularly sensitive to the signals that maintain survival or induce death, they are particularly susceptible to ferroptosis. Many factors that are conducive to tumor growth, such as iron accumulation, increased lipid synthesis, or EMT, can make tumor cells more sensitive to ferroptosis. A variety of chemotherapeutic agents also exert anti-tumor activity by inducing oxidative stress, interfering with the anabolism of genetic substances and other means, and exert a synergistic anti-cancer effect with ferroptosis. However, how to target tumor cells to induce ferroptosis is a tough problem at present. This is because excessive induction of ferroptosis usually causes certain damage to normal tissues. For example, iron deposition often occurs in the process of brain aging, mainly in the substantia nigra, globular nucleus, caudate nucleus, and cerebral cortex. Ferroptosis induced by deposited iron is often closely related to neurodegenerative diseases (Tang et al., 2021). Alzheimer’s disease (AD) is the most common neurodegenerative disease that causes cognitive dysfunction. The pathological characteristics of AD are the accumulation of lipid peroxides and the imbalance of iron homeostasis (Wang et al., 2019). GPX4 gene-deficient mice have the biochemical characteristics of ferroptosis and the performance of AD, and AD performance can be alleviated by ferroptosis inhibitors (Ward et al., 2014). Except for neurological diseases, ferroptosis also plays an important role in ischemia–reperfusion injury (Hu et al., 2019). Ferroptosis inhibitors have been used to treat intestinal injury and rhabdomyolysis caused by ischemia–reperfusion (Martin-Sanchez et al., 2017; Li et al., 2019). In addition, inhibitors of ferroptosis have good renal protection effect in the model of acute kidney injury (Skouta et al., 2014; Yan et al., 2020). Targeting ferroptosis in tumor cells to avoid the occurrence in normal tissues is of great significance for the precise treatment of tumors, and it is also one of the urgent problems that need to be solved in the current research field of ferroptosis.

Nano-vehicle agents may provide a solution for targeting tumor cell ferroptosis. Compared with traditional drugs, nano-vehicle agents can greatly improve the ability to selectively kill tumors and increase tumor treatment effects (Hussain et al., 2021). By carrying ferroptosis inducers on nano-vehicles, the solubility, biocompatibility, and tumor targeting can be greatly improved (Kim et al., 2016). For example, ferroptosis inducers and chemotherapeutic agents have been loaded into silica nanoparticles. These nanoparticles can attract iron in the extracellular environment, increase the iron content in tumor cells, and express ferritin *in vivo*. Inhibiting the expression of GSH will increase the level of intracellular ROS and promote ferroptosis (Ma et al., 2017). Iron can also be directly loaded into nanoparticles, such as ferric oxide nanoparticles or platinum–iron nanoparticles. When these nanoparticles reach the tumor site, they will release iron and promote ferroptosis of cells (Yue et al., 2018; Zhang et al., 2019). According to the mechanism of ferroptosis, nanoparticles also have various designs, which can act on mechanisms such as iron, lipid, or amino acid metabolism. For example, because the catalytic effect of Fe^2+^ is better that of than Fe^3+^, you can choose to directly increase the concentration of Fe^2+^ in the cell or carry Fe^3+^ and tannic acid at the same time, which can reduce Fe^3+^ to Fe^2+^, so that the cell will be maintained in the process of ferroptosis (Lin et al., 2018; Liu T. et al., 2018). In addition, the substrate required to produce ROS can be carried in nanoparticles, which directly increases the level of intracellular ROS, thereby improving the efficacy of the drug (Zheng et al., 2017). The mechanism of nano-vehicles in targeting tumors is related not only to the specific antigen expressed on tumors, but also to the difference between normal tissue and TME (Dai et al., 2017). Therefore, the future design of nano-vehicle agents should also focus on targeting the TME, and promoting ferroptosis in tumor cells by reshaping the TME may be able to bring a new strategy for tumor treatment.

## Conclusion

Ferroptosis is a novel type of RCD, and its mechanism and application are in a period of rapid development. The core mechanism is iron metabolism and lipid peroxides. The discovery of FSP1 as a negative regulator enriches the connotation of ferroptosis. The in-depth study of ferroptosis also provides more possible therapeutic targets for tumor treatment. New drugs and treatment methods for these targets are expected to be developed. At present, resistance to traditional chemotherapy, targeted therapy, or immunotherapy agents is the most critical factor for refractory tumor or recurrence. Due to the universality of the drug resistance mechanism, when tumor cells are resistant to a drug, they usually have different degrees of resistance to other drugs of the same type. A small number of tumor cells even appear multi-drug resistant, which greatly impairs the efficacy of the drug and shortens the survival time of patients. Many studies have confirmed that ferroptosis can promote tumor cell death through synergistic effects with anti-tumor drugs, improve drug efficacy, and even reverse drug resistance. It provides a new treatment strategy for patients with drug resistance. Compared with normal cells, the dependence of tumor cells on iron makes them more sensitive to iron load and ROS accumulation. Therefore, targeting iron ions is a feasible method that can improve the efficacy and reduce the side effects of treatment. Cross-field technologies can be combined. For example, the combination of nano-vehicle agents and ferroptosis may be more suitable for tumor treatment. However, before clinical application, many problems still need to be solved. For example, although many methods can be used to detect the level of ferroptosis *in vitro*, better methods for evaluating ferroptosis *in vivo* are still lacking. Without sensitive and accurate detection indicators, the drug’s efficacy cannot be accurately detected leading to risks and injuries caused by treatment. Second, whether the compound targeted to the ferroptosis regulator can maintain a high degree of specificity and minimize side effects in preclinical or clinical applications must be clarified. Finally, which types or characteristics of tumors, such as tumors with certain gene mutations, are more susceptible to ferroptosis remains to be elucidated. However, it is undeniable that in-depth studies of the mechanism of ferroptosis and tumor drug resistance will surely create new opportunities for tumor diagnosis and treatment.
